# Causal Relationships Between Dietary Habits, Gut Microbiota, Metabolites, Serum Proteins and Laboratory Biomarkers in Kidney Stone Formation: A Mendelian Randomisation Study

**DOI:** 10.1111/jcmm.70698

**Published:** 2025-07-17

**Authors:** Rongjiang Wang, Mengting Jiang, Zhaojun Li, Changbao Xu, Hui Liang, Junwen Shen, Huan Zhong

**Affiliations:** ^1^ The Department of Urology The First Afliated Hospital of Huzhou Normal College Zhejiang China; ^2^ Huzhou Key Laboratory of Precise Diagnosis and Treatment of Urinary Tumors Zhejiang China; ^3^ The Department of Urology The Second Affiliated Hospital of Zhengzhou University Zhengzhou Henan China; ^4^ The Department of Urology Xinchang County Traditional Chinese Medicine Hospital Zhejiang China

**Keywords:** dietary habits, gut microbiota, kidney stone, Mendelian randomisation, metabolites and biomarkers

## Abstract

This study aimed to elucidate the causal interplay between dietary habits, gut microbiota composition, circulating metabolites, serum proteins, laboratory biomarkers and kidney stone formation, employing Mendelian randomisation (MR) to identify potential mediators. A rigorous two‐sample MR framework was employed to assess the causal associations between kidney stones and a spectrum of predisposing factors. This encompassed dietary patterns, gut microbiota profiles, circulating metabolic intermediates, serum proteins and laboratory test indicators. Significant associations were further analysed using mediation analysis to uncover indirect pathways. Initial significance was determined at *p* < 0.05, followed by the implementation of False Discovery Rate correction (FDR *p* < 0.05) to reduce the likelihood of false positives due to multiple comparisons. Direct causal relationships were established between kidney stones and 9 dietary factors (including fruit, alcohol, coffee intake), 11 gut microbiota types, 8 metabolites, 12 plasma proteins and 8 laboratory indicators (CRE, EGFR, CA, UAHDL, APOA, CYS and URNA). Notably, nine mediation pathways were discovered. These pathways reveal the indirect effects of dietary habits on kidney stone formation mediated through laboratory biomarkers. Specifically, five dietary habits—alcohol, coffee, fruit, champagne/white wine and dried fruit consumption—were shown to mediate through seven key factors: APOA, CA, CYS, EGFR, HDL, UA and URNA. Six of these mediations were positive, indicating facilitatory roles, while three exhibited negative mediation, suggestive of competitive inhibition in the diet‐kidney stone causal pathway. This MR study underscored the causal links between dietary habits, gut microbiota composition, circulating metabolites, serum proteins, laboratory biomarkers and kidney stone development, shedding light on potential mediators including seven laboratory biomarkers.

## 1 Background

Kidney stones represent a major global health concern. They significantly impact public well‐being due to their high occurrence and frequent recurrence. To begin with, data from the National Institute of Diabetes and Digestive and Kidney Diseases (NIDDK) in the United States show a significant gender difference in the lifetime occurrence of kidney stones among adults, affecting about 11.3% of men and 6.3% of women [1]. In Europe, the estimated lifetime prevalence of kidney stones ranges from 5% to 9%, with Germany notably approaching the 10% threshold [2]. In China, according to the Chinese Medical Tribune, the incidence of kidney stones among adults falls within the range of 8% to 10%, with significantly higher rates in southern regions, reaching 12% to 13% [3]. Secondly, concerning high recurrence rates, multiple international studies have summarised that the recurrence rate within 5 years after kidney stone diagnosis ranges between 30% and 50%, while long‐term observations spanning 10 years have revealed an alarming increase to 60%–80% [4, 5].

Despite significant progress in kidney stone research over the past few decades, significant challenges remain in establishing core conclusions for prevention. The primary obstacle lies in the complex interplay of numerous clinical factors, known as the ‘confounding effect’, which involve factors such as dietary habits, gut microbiota, serum metabolites and plasma protein levels. These factors interact with each other, making it difficult for researchers to accurately discern the direct and indirect roles they play in kidney stone formation. Consequently, developing universally recognised and standardised prevention guidelines has proven to be especially challenging.

To overcome these research bottlenecks, our team has innovatively employed Mendelian randomisation (MR) methodology to delve into the pathogenesis of kidney stones. By utilising genetic variations as natural instrumental variables, MR studies effectively circumvent the confounding factors prevalent in traditional observational studies, providing a powerful tool for precisely disentangling the intricate relationships among key clinical factors [6, 7].

Our team embarked on an investigation from five critical dimensions: dietary habits, gut microbiota composition, serum circulating metabolite levels, plasma protein profiles and laboratory test indicators. We combined seven extensive Genome‐Wide Association Study (GWAS) data sets, involving approximately 900,000 individuals from four different nations. Leveraging a two‐sample MR analysis framework and a two‐step mediation MR study design, we successfully unveiled direct causal relationships between 9 dietary factors, 11 gut microbiota types, nine metabolites, 12 plasma proteins and 8 laboratory indicators with kidney stone formation. Notably, we also discovered nine sets of mediation pathways involving specific dietary habits indirectly influencing kidney stone development through laboratory test items, offering novel insights and scientific evidence for a deeper understanding of kidney stone pathogenesis.

## 2 Methods

### 2.1 Study Design

**FIGURE 1:**
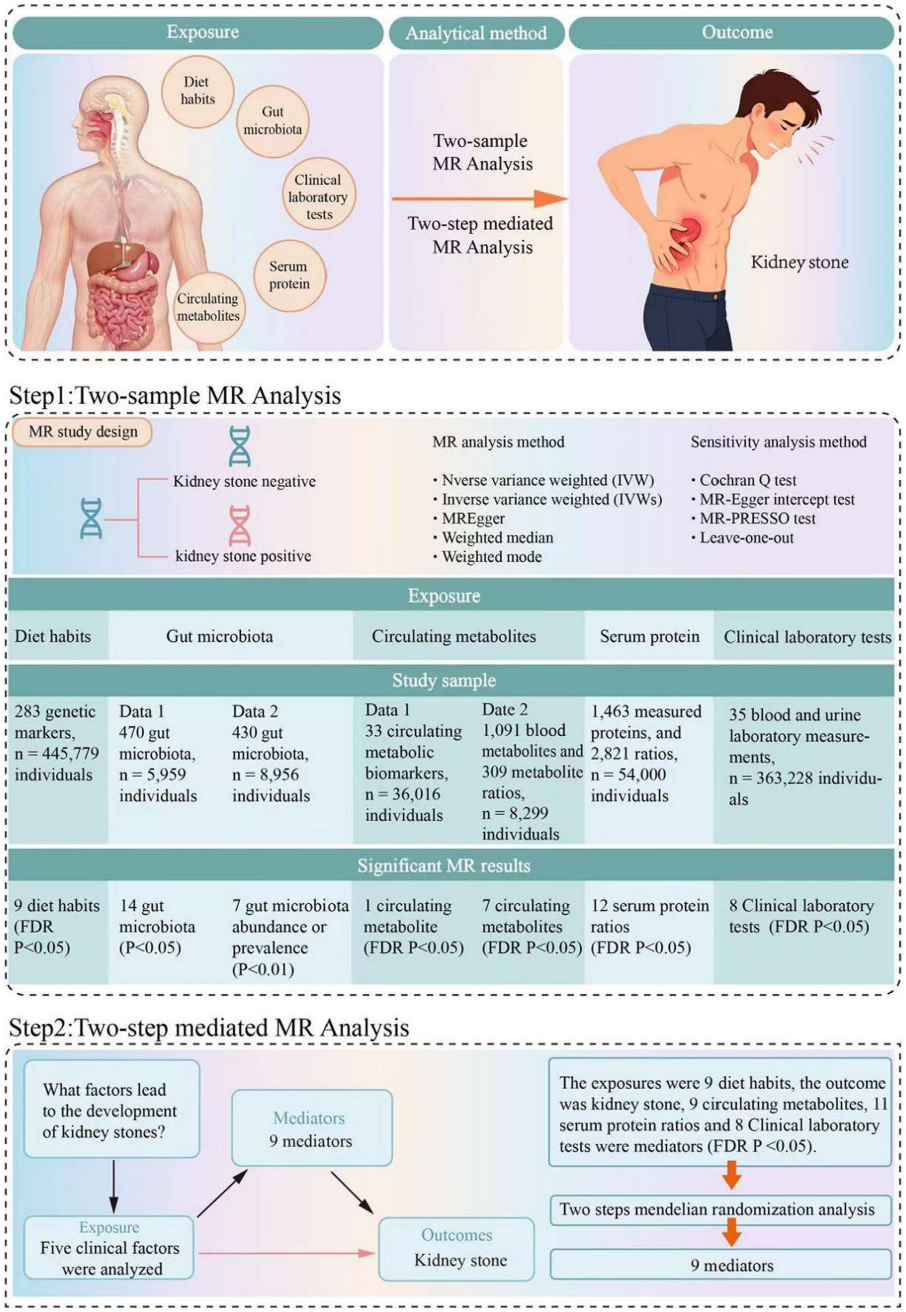
Study design: A rigorous two‐sample MR framework was employed to assess the causal associations between kidney stones and a spectrum of predisposing factors. This encompassed dietary patterns, gut microbiota profiles, circulating metabolic intermediates, serum proteins and laboratory test indicators.

We performed an extensive study on kidney stones and several possible contributing factors, such as diet, gut bacteria, blood metabolites, serum proteins and lab test indicators, utilising a two‐sample Mendelian randomisation approach, followed by mediation analysis on numerous significant findings. Figure 1 provides a summary of the research design. For this observational research, the STROBE‐MR (Strengthening the Reporting of Observational Studies in Epidemiology using Mendelian Randomisation) checklist was filled out and included in the Supporting Information.

### 2.2 Data Sources

In order to elucidate the possible causes of kidney stones, this research incorporated GWAS data from various origins: [1] diet's data sources were described from a cohort of 445,779 UK Biobank participants, which identified 283 genetic markers associated with 38 diet habits [8], [2] two important gut microbiota's data sources were enrolled. Data 1 originated from a group of 5959 Finnish participants, encompassing 2801 microbial species and 7,967,866 genetic variations in humans [9]. Data 2 originated from a group of 8956 German participants, identifying 38 genetic locations linked to individual bacteria and the overall microbiome structure [10], [3] circulating metabolites' sources also contained two huge data. Data 1 came from a genome‐wide characterisation of circulating metabolic biomarkers which was published in Nature in 2022, which quantified 233 circulating metabolic biomarkers and identified more than 400 independent loci from 36,016 participants from 33 cohorts [11]. A year later, Nature Genetics published findings data 2, which included a greater number of metabolic biomarkers: 1091 blood metabolites and 309 metabolite ratios [12], [4] the data for serum proteins originated from a large‐scale genetic association study, which included measurements of 1463 proteins and 2821 protein level ratios, derived from more than 54,000 samples in the UK Biobank [13], [5] the relationship between clinical lab tests and kidney stones was assessed, utilising genetic data from 35 blood and urine measurements in the UK Biobank, involving 363,228 participants [14] and [6] renal stone data sources were from finngen_R10_N14_CALCUKIDUR [15]. The above data are all public and have been downloaded by us for subsequent analysis.

### 2.3 Data Extraction

We applied a set of selection criteria to identify suitable genetic IVs. We opted for a fairly strict threshold (*p* < 1 × 10^–8^) to capture potential variant sets enriched for association and achieve thorough results. We performed a clumping process (*R* ^2^ < 0.001, window size = 10,000 kb) to remove variants with high linkage disequilibrium (LD) and guarantee the independence of each SNP. Single nucleotide polymorphisms (SNPs) with a minor allele frequency below 0.01, those with unclear alleles, and palindromic SNPs were omitted [16, 17].

### 2.4 Genetic Studies to Determine Causation

Initially, we performed one‐way MR analyses to investigate the causal link between kidney stones and several possible predisposing factors. Five MR techniques were utilised: inverse variance weighted (IVW), inverse variance weighted with multiplicative random effects (IVWs), MR‐Egger, weighted median and weighted mode. The IVW technique is regarded as the most precise and robust approach. It is used for determining causal effects when all chosen SNPs are legitimate instrumental variables. The traditional MR technique, inverse variance‐weighted (IVW) method, was employed to estimate effects, presented as beta (β) values with standard errors for continuous outcomes and odds ratios (OR) with 95% confidence intervals (CI) for binary outcomes; *p* values less than 0.05 were deemed nominally significant. Furthermore, the false discovery rate (FDR) adjustment was utilised in later analyses to minimise the chance of false positives due to the concurrent testing of several hypotheses. An FDR value below 0.05 was deemed highly significant [18, 19].

### 2.5 Mediation MR Analysis

This research considered various causes of kidney stones, highlighting diet as a primary influence on gut microbiota, blood metabolites, plasma proteins and lab results. Therefore, the following two‐step Mendelian randomisation (MR) analysis was mainly conducted to investigate the mediating roles of dietary patterns and kidney stones, taking into account gut microbiota, blood metabolites, plasma proteins and lab tests as intermediary factors in diet‐related kidney stone formation. Potential dietary habits, circulating metabolites, plasma proteins and laboratory tests that met the inclusion criterion of FDR *p* < 0.05 in the previous MR analysis were selected as elements for subsequent analysis.

Initially, the causal impact of the diet on the mediator (β1) was assessed using Mendelian randomisation. The next phase involved assessing the causal impact of each mediator on kidney stones through MR, adjusting for dietary factors (β2), based on the assumption that the mediator had a causal link to kidney stones in MR. The mediation share of each intermediary in the link between diet and kidney stones was determined by multiplying β1 and β2, then dividing by the overall impact of diet on kidney stones. The 95% confidence intervals for the mediation proportions were determined through the delta method [20].

### 2.6 Sensitivity Analyses

In our study, we employed a comprehensive approach involving up to four Mendelian randomisation (MR) methodologies—specifically, MR‐Egger, weighted median, simple mode and weighted mode—each underpinned by distinct pleiotropy assumptions, to generate robust effect estimates as part of our sensitivity analyses. To interrogate horizontal pleiotropy, we leveraged the MR‐Egger technique, which applies a weighted linear regression framework with an unconstrained intercept. This intercept serves as a proxy for the average pleiotropic influence across the studied genetic variants, reflecting the mean direct effect a variant exerts on the outcome of interest. Notably, the deviation of the intercept from zero, as indicated by a statistically significant MR‐Egger intercept *p* value (< 0.05), constituted evidence of horizontal pleiotropy.

Furthermore, we assessed heterogeneity among the estimates using Cochrane's Q test, where decreasing *p* values suggest elevated heterogeneity and a heightened likelihood of directional pleiotropy. To identify potential outliers at the single‐nucleotide polymorphism (SNP) level, we conducted leave‐one‐out analyses. The sensitivity analysis of all significant findings was subsequently presented in an appendix, ensuring transparency and reproducibility of our results [21].

All Mendelian randomisation studies were performed in R utilising the packages ‘TwoSampleMR’, ‘tidyverse’, ‘ggplot2’, ‘purrr’, ‘data.table’ and ‘LDlinkR’. False discovery rate (FDR) *p* values were calculated with the R package ‘p.adjust’, while the linkage disequilibrium score regression (LDSC) was performed using the LDSC tool in Python (version 3.10.5; https://www.python.org/) [22].

### 2.7 Authorisation and Agreement for Involvement

This research utilises data that are accessible to the public. Each study in the GWAS was sanctioned by the appropriate Institutional Review Board, and participants provided informed consent from the participants themselves or a caregiver, legal guardian or another representative.

## 3 Results

### 3.1 Genetic Causality and Correlation Between Diet Habits and Kidney Stone

**FIGURE 2:**
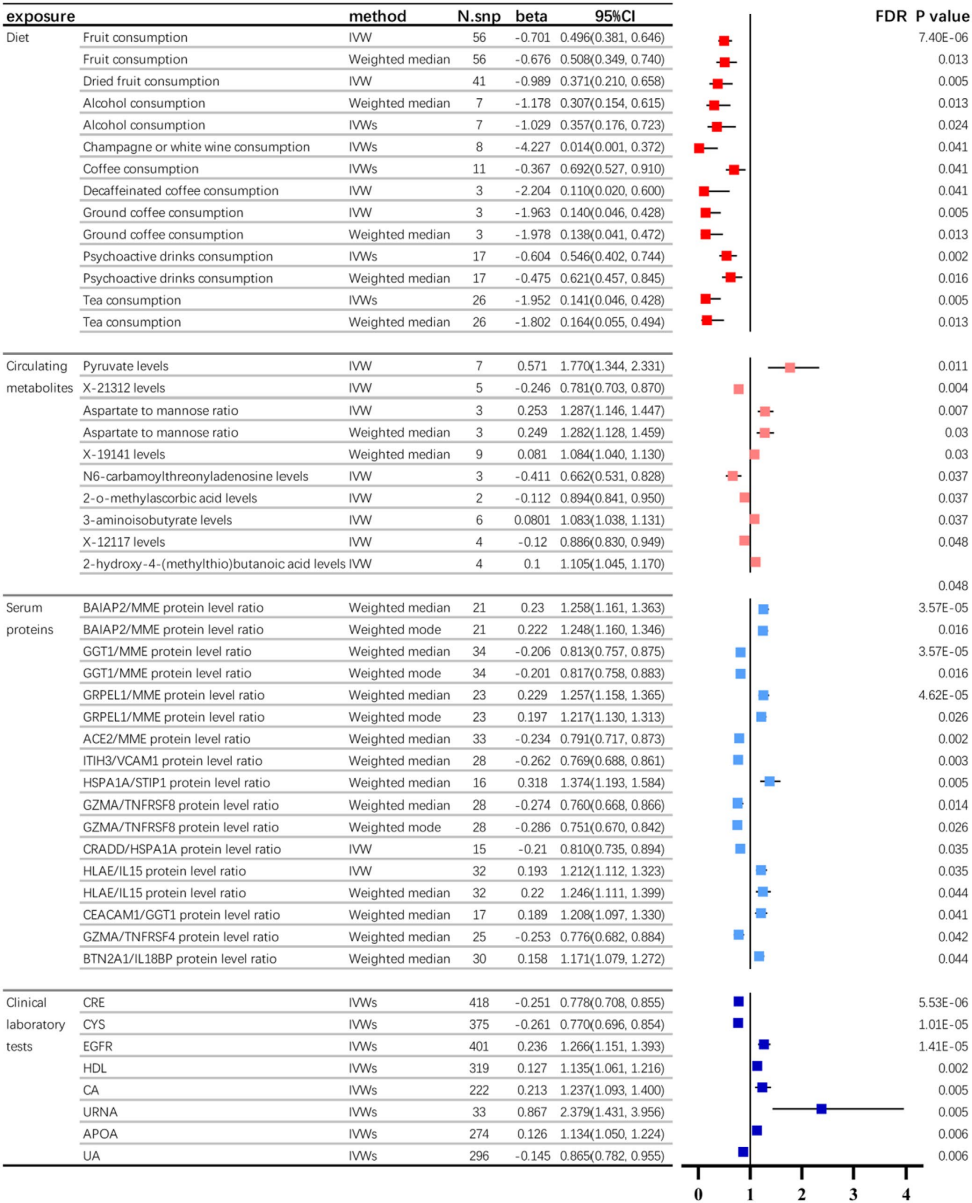
Genetic causality and correlation between dietary habits, circulating metabolites, serum proteins, clinical laboratory tests and kidney stone by two sample MR analysis. In this study, we observed (a) alcohol, psychoactive drinks, coffee and tea consumption exhibited significant inverse associations with kidney stones, indicated by MR analyses (β < −1); (b) eight metabolites and one metabolite ratio were identified as relevant to kidney stone formation (FDR *p* < 0.05). Elevated N6‐carbamoylthreonyladenosine, X‐21312, X‐12117 and 2‐o‐methylascorbic acid were protective, whereas increased Pyruvate, Aspartate/Mannose ratio, X‐19141, 3‐aminoisobutyrate and 2‐hydroxy‐4‐(methylthio)butanoic acid were risk factors; (c) twelve plasma protein ratio pairs were significantly linked to kidney stones (FDR *p* < 0.05), with five (BAIAP2/MME, GRPEL1/MME, GGT1/MME, GZMA/TNFRSF8 and HLAE/IL15) confirmed by multiple MR methods. Five promoted stone formation, whereas six inhibited it; (d) Eight blood test indicators (CRE, EGFR, CA, UAHDL, APOA, CYS and URNA) were closely associated with kidney stones (FDR *p* < 0.05), with CRE, CYS and UA exhibiting protective effects and higher EGFR, CA, HDL, APOA and URNA levels contributing to stone development. Part labels (a–d) represent the four sections of the table.

In assessing the impact of 38 different dietary habits on kidney stones, we found that one sequence and nine specific habits have a negative correlation with kidney stones. These habits include eating fruit, drinking alcohol, consuming coffee, psychoactive beverages and tea. Among them, multiple MR analysis methods have indicated that alcohol consumption, psychoactive drink consumption, coffee consumption and tea drinking show a more significant protective correlation with kidney stones. The beta value is less than −1. Among them, long‐term alcohol intake shows the most significant preventive effect on kidney stones, with both IVW and weighted median analyses supporting its protective role against kidney stone formation (IVW FDR *p* value = 0.02, weighted median FDR *p* value = 0.01). Additionally, champagne or white wine consumption emerges as the best protective factor for kidney stones. The beta value is −4.2, and the IVW FDR *p* value is 0.04. Long‐term consumption of various beverages is also a protective factor for kidney stones. This research discovered that prolonged consumption of coffee, whether regular, decaffeinated or ground, proved advantageous for people with kidney stones (FDR *p* < 0.05). Additionally, extended tea drinking was beneficial for this group (IVW FDR *p* < 0.01, Weighted median FDR *p* = 0.013). Even long‐term consumption of addictive beverages was able to reduce the occurrence of kidney stones (IVWs FDR *p* < 0.01, Weighted median FDR *p* = 0.016, Figure 2).

### 3.2 Genetic Links and Associations Between Intestinal Microbiome and Renal Calculi

Firstly, regarding the two large‐scale gut microbiota data sets (Finnish data set and German data set), if we adopt FDR *p* < 0.05 as the strict screening criterion, we fail to obtain any positive results that can be used for subsequent analysis. Secondly, with *p* < 0.05 as the screening criterion, we found a series of positive results from the two gut microbiota data sets.

In the German gut microbiota study, we identified a total of 14 potentially important gut microbiota that have a causal relationship with kidney stone formation. Among these, 11 gut microbiota have a unidirectional causal relationship with kidney stones (either in the direction of prevalence or abundance), and 3 left gut microbiota have a bidirectional causal relationship with kidney stones (both in the direction of prevalence and abundance). Upon analysing the positive and negative associations of beta values for the presence or absence of certain bacteria using various MR analysis tools, we discovered that just three bacteria consistently showed a causal link to the formation of kidney stones. Alistipes (beta = −0.10, *p* = 0.011) and Parabacteroides (beta = −0.03, *p* = 0.043) serve as protective agents against kidney stone formation, whereas Prevotella (beta = 0.02, *p* = 0.007) significantly contributes to the risk of developing kidney stones.

**FIGURE 3:**
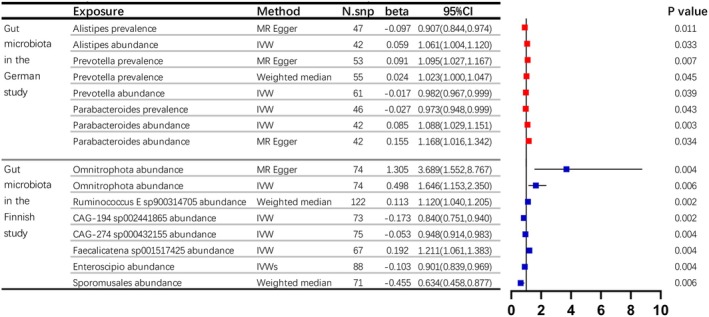
Genetic links and associations between intestinal microbiome and renal calculi by two‐sample MR analysis. In this study, we highlight (a) in the German gut microbiota cohort, three microbiota species exhibited bidirectional causality with kidney stones. Alistipes (β = −0.10, *p* = 0.011) and Parabacteroides (β = −0.03, *p* = 0.043) were protective, whereas Prevotella (β = 0.02, *p* = 0.007) increased risk; (b) the Finnish data set revealed seven species with stronger causal associations (*p* < 0.01). Omnitrophota, Ruminococcus E sp900314705 and Faecalicatena sp001517425 were protective, whereas CAG‐194 sp002441865, CAG‐274 sp000432155, Enteroscipio and Sporomusales significantly contributed to kidney stone development. Part labels (a‐b) represent the two sections of the table.

Compared to the German data set, the Finnish data set exhibits two main differences. Firstly, it contains more information on gut microbiota. Secondly, it only presents information on the absence of specific cells but lacks information on the presence of specific bacteria. Within the Finnish data set, we discovered 58 gut microbiota species with significant findings (*p* < 0.05), and among these, 7 species exhibited a stronger causal link to kidney stone formation (*p* < 0.01). Omnitrophota, Ruminococcus E sp900314705 and Faecalicatena sp001517425 serve as protective agents against kidney stone formation, whereas CAG‐194 sp002441865, CAG‐274 sp000432155, Enteroscipio and Sporomusales act as pathogenic contributors to kidney stone development (Figure 3).

### 3.3 Genetic Causality and Correlation Between Circulating Metabolites and Kidney Stone

Based on two plasma metabolome data sets, we identified eight significant metabolites and one metabolite ratio directly associated with the onset of kidney stones (all FDR *p* < 0.05), with one from data 1 and the remaining seven from data 2. Elevated levels of four metabolites were protective factors against kidney stone formation, namely N6‐carbamoylthreonyladenosine levels (β = −0.41), X‐21312 levels (β = −0.24), X‐12117 levels (β = −0.12) and 2‐o‐methylascorbic acid levels (β = −0.11). Furthermore, higher concentrations of four blood metabolites and one metabolite ratio were linked to an increased risk of kidney stones, namely Pyruvate (β = 0.57), Aspartate to mannose ratio (β = 0.25), X‐19141 (β = 0.08), 3‐aminoisobutyrate (β = 0.08) and 2‐hydroxy‐4‐(methylthio)butanoic acid (β = 0.1).

### 3.4 Genetic Causality and Correlation Between Serum Proteins and Kidney Stone

We employed what is likely the most extensive data set of plasma proteins so far (including 1463 proteins and 2821 protein level ratios), which was used to investigate the causal links between plasma protein levels and the occurrence of kidney stones. The results suggest significant associations between 12 pairs of plasma protein ratios and kidney stone occurrence (FDR *p* < 0.05). Among them, five protein ratio pairs (BAIAP2/MME protein level ratio, GRPEL1/MME protein level ratio, GGT1/MME protein level ratio, GZMA/TNFRSF8 protein level ratio and HLAE/IL15 protein level ratio) were supported by findings from two distinct MR analysis methods. Five of these protein ratio pairs positively promote kidney stone development: BAIAP2/MME protein level ratio, GRPEL1/MME protein level ratio, HLAE/IL15 protein level ratio, CEACAM1/GGT1 protein level ratio and BTN2A1/IL18BP protein level ratio. Concurrently, six protein ratio pairs were found to inhibit kidney stone occurrence: GGT1/MME protein level ratio, ACE2/MME protein level ratio, ITIH3/VCAM1 protein level ratio, GZMA/TNFRSF8 protein level ratio, CRADD/HSPA1A protein level ratio and GZMA/TNFRSF4 protein level ratio.

### 3.5 Genetic Causality and Correlation Between Clinical Laboratory Tests and Kidney Stone

We further conducted a causal analysis on the common 35 urinary and blood test indicators in relation to the onset of kidney stones. The results suggest that a total of eight test indicators are closely associated with the incidence of kidney stones (FDR *p* value < 0.05), including eight blood test indicators (CRE, EGFR, CA, UAHDL, APOA, CYS and URNA). CRE, CYS and UA help prevent kidney stones, whereas higher concentrations of EGFR, CA, HDL, APOA and URNA contribute to their formation. It is important to highlight that the connection between the eight urine and blood test markers and kidney stones holds greater clinical relevance (all *p* < 0.01).

### 3.6 Mediators Between the Diet and the Kidney Stone

In this study, we have included nine important dietary habits that have been confirmed in the early stage, including alcohol consumption, champagne or white wine consumption, coffee consumption, decaffeinated coffee consumption, dried fruit consumption, fruit consumption, ground coffee consumption, psychoactive drinks consumption and tea consumption. Since the results of gut microbiota did not meet the criteria of FDR *p* value < 0.05, they were not included in the subsequent mediation analysis. Additionally, some circulating metabolites and plasma protein SNPs failed to meet the requirements of all three databases involved in the two‐step mediation method. In the end, the subsequent analysis included the following mediators: eight circulating metabolites (Pyruvate, X‐21312, Aspartate to mannose ratio, N6‐carbamoylthreonyladenosine, 2‐o‐methylascorbic acid, 3‐aminoisobutyrate, X‐12117 and 2‐hydroxy‐4‐(methylthio)butanoic acid), two plasma protein ratios (CRADD/HSPA1A and HLAE/IL15) and eight laboratory test indicators (CRE, CYS, EGFR, HDL, CA, URNA, APOA and UA).

**FIGURE 4:**
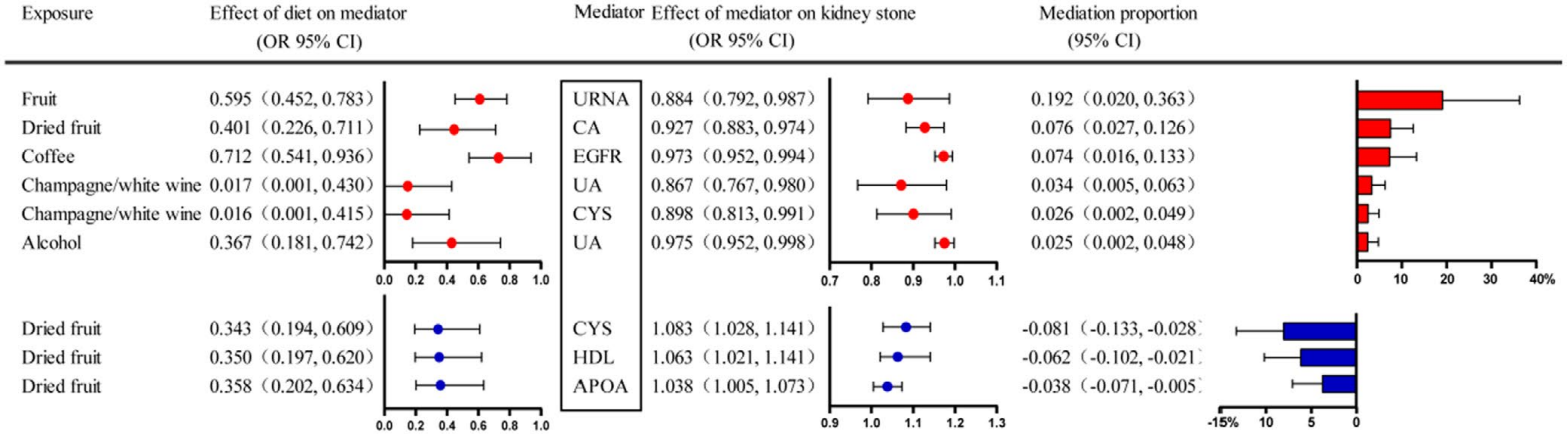
Mediating role of each mediator in the causal association between the diet and the kidney stone. This figure illustrates the interplay between five dietary habits and kidney stone formation, mediated by seven key biochemical factors (APOA, CA, CYS, EGFR, HDL, UA and URNA). Notably, six positive mediating relationships indicate a direct influence of specific dietary patterns (alcohol, coffee, fruit, champagne/white wine and dried fruit consumption) on kidney stone development via elevated lab markers. Conversely, three negative mediating pathways suggest a mitigating or competitive role in this causal chain, highlighting the complex interplay between diet and biochemical mediators in kidney stone pathogenesis.

In the two‐step MR analysis, with the condition that both OR analyses maintained consistent positive or negative values, nine positive mediating outcomes were ultimately selected. These comprised five dietary habits (Alcohol consumption, Coffee consumption, Fruit consumption, Champagne or white wine consumption and Dried fruit consumption), along with seven key mediating factors (APOA, CA, CYS, EGFR, HDL, UA and URNA). Among these, six mediating relationships were positive, indicating that specific dietary habits influenced the occurrence of kidney stones through specific laboratory indicators. There were also three mediating relationships with negative values, suggesting that these specific mediating relationships had a competitive effect on the causal link between diet‐induced kidney stones. Notably, the most significant mediating relationship was observed between Fruit consumption and the incidence of kidney stones, with urinary URNA serving as the mediating factor (with a mediation proportion as high as 19.2%), exhibiting a protective effect against kidney stone formation (Figure 4).

## 4 Discussion

In our exploration of the multifaceted factors contributing to kidney stone formation, dietary habits emerged as the most prominent influence, as identified by this study. Nine dietary practices were systematically recognised, all exhibiting significant protective effects against kidney stone development. Notably, moderate consumption of champagne or white wine was confirmed as one of the most effective dietary strategies for kidney stone prevention. Furthermore, this study categorically included coffee, addictive beverages and tea as positive factors in preventing kidney stone formation, a stark contrast to previous assumptions that these drinks, due to their oxalate content, might increase kidney stone risk [23, 24].

Previous research often focused on the potential negative effects of oxalate in coffee and tea, suggesting long‐term intake could elevate urine oxalate levels, thereby promoting kidney stone formation [24]. However, our rigorous data analysis conclusively demonstrated that long‐term moderate consumption of coffee, tea, addictive beverages and champagne‐type alcoholic drinks is actually effective in preventing kidney stones. We speculate that these dietary habits exert their protective effects primarily through their positive impact on daily urine output. Studies have highlighted the association between moderate alcohol consumption (especially beer) and reduced kidney stone risk, with weekly moderate beer consumption associated with a 41% lower risk [25]. Similarly, caffeine, a natural diuretic, promotes urine production and excretion, diluting urine mineral concentrations and thereby reducing conditions conducive to stone formation. Furthermore, we hypothesise that the addictive nature of these beverages may encourage sustained, relatively frequent consumption patterns in specific populations, ensuring continuous diuretic effects and serving as a key mechanism in kidney stone prevention.

Our in‐depth research unveiled significant causal associations between eight clinical biomarkers and kidney stone development. This finding provides crucial insights for clinical practice. These biomarkers encompass key renal function parameters (e.g., CRE, EGFR), indicators of serum uric acid homeostasis (URNA and UA), serum calcium concentration (CA), lipid metabolism‐related markers such as HDL and ApoA and the amino acid Cys, which has been less frequently linked to kidney stone prevention in prior studies.

Notably, the significance of traditional high‐risk factors was reconfirmed in our study. These factors include renal function status [26], abnormal serum uric acid levels [27] and high serum calcium [28] as contributors to kidney stone formation, which was reconfirmed in our study, aligning with previous findings. However, our study also uniquely identified HDL and ApoA as emerging facilitators of kidney stone development, challenging existing understandings and suggesting a novel role for lipid metabolism abnormalities in kidney stone pathogenesis. Additionally, unexpectedly, elevated Cys levels emerged as a protective factor against kidney stones, offering a new perspective for kidney stone prevention strategies.

By employing a two‐step Mendelian randomisation (MR) approach, we pinpointed particular dietary habits that influence kidney stone formation via seven identified biomarkers [29]. This finding improves our grasp of the intricate relationship between nutrition and kidney stone development, allowing for more accurate forecasting and customisation of eating patterns through consistent tracking of crucial biomarkers, thus establishing a robust basis for personalised medicine in preventing kidney stones.

Comprehensively, we systematically identified potential causal relationships between 11 gut microbiota profiles, 9 circulating metabolites and 12 plasma protein ratios with kidney stone development. Notably, most of these identified factors are being reported for the first time in significant association with kidney stone disease, significantly broadening our understanding of its pathophysiological mechanisms. While these clinical factors exhibited associations with kidney stone development, their individual direct effects on disease perturbation did not dominate [30].

In analysing the relative contributions of various factors to kidney stone prevention, we found that dietary modifications were particularly impactful, emphasising the central role of lifestyle interventions in kidney stone management. From a practical and economic perspective, the eight clinical biomarkers we identified are not only easy to implement but also cost‐effective, providing convenient and efficient tools for clinical decision‐making.

However, it is crucial to note that relying solely on specific dietary changes or monitoring a few biomarkers may fall short in comprehensively capturing the dynamic changes and individual differences within the kidney stone patient population. Thus, we underscore the importance of developing a multidimensional, refined kidney stone risk assessment system based on plasma proteomics, micro‐circulating metabolites and other approaches to achieve more precise disease prediction and management. Furthermore, the specific mechanisms by which gut microbiota influence kidney stone formation remain to be explored in larger, multi‐layered studies.

## 5 Conclusion

In a pioneering study, we comprehensively assessed the causal relationships between dietary habits, gut microbiota, serum metabolites, plasma proteins, laboratory indicators and kidney stone formation. We identified direct causal links involving 9 dietary habits, 9 gut microbiota types, 8 metabolites, 12 plasma proteins and 8 laboratory items. Notably, we also discovered nine mediation pathways, whereby dietary habits indirectly influence kidney stones through laboratory indicators. These discoveries enhance our comprehension of the causes of kidney stones and indicate possible treatment targets.

## Author Contributions

**Rongjiang Wang:** writing – original draft (equal). **Mengting Jiang:** conceptualization (equal). **Zhaojun Li:** conceptualization (equal). **Changbao Xu:** conceptualization (equal). **Hui Liang:** conceptualization (equal). **Junwen Shen:** conceptualization (equal). **Huan Zhong:** conceptualization (equal).

## Ethics Statement

This research utilises data that are accessible to the public. Each study in the GWAS was sanctioned by the appropriate Institutional Review Board, and participants provided informed consent from the participants themselves or a caregiver, legal guardian or another representative.

## Conflicts of Interest

The authors declare no conflicts of interest.

## Data Availability

The original data presented in this study have been included in this article. All data were retrieved from public databases.
